# Objective User Engagement With Mental Health Apps: Systematic Search and Panel-Based Usage Analysis

**DOI:** 10.2196/14567

**Published:** 2019-09-25

**Authors:** Amit Baumel, Frederick Muench, Stav Edan, John M Kane

**Affiliations:** 1 Department of Community Mental Health University of Haifa Haifa Israel; 2 The Partnership for Drug-Free Kids New York, NY United States; 3 Psychiatry Research, The Zucker Hillside Hospital, Northwell Health Glen Oaks, NY United States

**Keywords:** user engagement, usage, adherence, retention, mental health, depression, anxiety, mHealth

## Abstract

**Background:**

Understanding patterns of real-world usage of mental health apps is key to maximizing their potential to increase public self-management of care. Although developer-led studies have published results on the use of mental health apps in real-world settings, no study yet has systematically examined usage patterns of a large sample of mental health apps relying on independently collected data.

**Objective:**

Our aim is to present real-world objective data on user engagement with popular mental health apps.

**Methods:**

A systematic engine search was conducted using Google Play to identify Android apps with 10,000 installs or more targeting anxiety, depression, or emotional well-being. Coding of apps included primary incorporated techniques and mental health focus. Behavioral data on real-world usage were obtained from a panel that provides aggregated nonpersonal information on user engagement with mobile apps.

**Results:**

In total, 93 apps met the inclusion criteria (installs: median 100,000, IQR 90,000). The median percentage of daily active users (open rate) was 4.0% (IQR 4.7%) with a difference between trackers (median 6.3%, IQR 10.2%) and peer-support apps (median 17.0%) versus breathing exercise apps (median 1.6%, IQR 1.6%; all *z*≥3.42, all *P*<.001). Among active users, daily minutes of use were significantly higher for mindfulness/meditation (median 21.47, IQR 15.00) and peer support (median 35.08, n=2) apps than for apps incorporating other techniques (tracker, breathing exercise, psychoeducation: medians range 3.53-8.32; all *z*≥2.11, all *P*<.05). The medians of app 15-day and 30-day retention rates were 3.9% (IQR 10.3%) and 3.3% (IQR 6.2%), respectively. On day 30, peer support (median 8.9%, n=2), mindfulness/meditation (median 4.7%, IQR 6.2%), and tracker apps (median 6.1%, IQR 20.4%) had significantly higher retention rates than breathing exercise apps (median 0.0%, IQR 0.0%; all *z*≥2.18, all *P*≤.04). The pattern of daily use presented a descriptive peak toward the evening for apps incorporating most techniques (tracker, psychoeducation, and peer support) except mindfulness/meditation, which exhibited two peaks (morning and night).

**Conclusions:**

Although the number of app installs and daily active minutes of use may seem high, only a small portion of users actually used the apps for a long period of time. More studies using different datasets are needed to understand this phenomenon and the ways in which users self-manage their condition in real-world settings.

## Introduction

The wide dissemination of mobile phone devices and the leap in the development and distribution of mobile health (mHealth) apps have altered the ways in which scholars conceptualize care management in the behavioral health domain. The conversation has shifted from patients and providers to individuals who can now engage in self-care around the clock outside of traditional health care settings (eg, [1-3]). Approximately 77% of the US adult population, and more than 89% of those younger than 50 years, now own a mobile phone [4,5] where they can store and use computerized apps. This widespread use has established a market for mHealth apps. Accordingly, a 2015 World Health Organization survey identified approximately 15,000 mobile apps for health care, with at least 29% designed for mental health [6].

The use of unguided apps has the potential to increase access to care in a scalable manner by reducing the costs associated with service uptake [7,8]. However, the impact of digital interventions is limited by their ability to engage users in therapeutic activities and to support user adherence to the therapeutic process [9,10]. Digital interventions require individuals to engage with self-care outside of traditional settings; therefore, individuals’ engagement must compete with other events in their daily lives and endure fluctuating motivation to be involved in effortful behavior [11]. As a result, user engagement with mobile apps and websites across the behavior change spectrum is low in the absence of human support [12-14]. Furthermore, various studies have suggested that most users of unguided Web-based programs exit websites before the full completion of the offered program [9,10,15,16]. For example, Christensen and colleagues [17] reported that less than 1% of users completed all modules in MoodGym, an open-access website for depression. In a systematic review of published articles reporting real-world user engagement with unguided programs for depression, anxiety, or mood enhancement, Fleming and colleagues [18] reported that 7% to 42% of users of Web- and app-based programs engaged in moderate use (completing between 40% and 60% of modular fixed-length programs or continuing to use the app after 4 weeks). For example, the developers of the PTSD Coach mobile app reported a usage decline over time, with 41.6% continuing to use the app 1 month after installation and 19.4% after 6 months [19]. Among Happify mobile app users, 3.5% completed a 6-week assessment. However, the authors noted that these users might have completed assessments without engaging in other content [20] (see [18] for a review).

Understanding patterns of real-world usage of e-mental health apps outside of empirical trials is key to maximizing the potential of apps to increase the public self-management of care. Utilization in real-world settings may differ from that in study settings for several reasons. First, empirical study settings include enrollment and assessment procedures that are not part of real-world utilization of the app, as trials largely emphasize internal validity over real-world generalizability [13]. Ebert and Baumeister [21] claim, for example, that within randomized trials “the securing of commitment represents an adherence-promoting element in self-help interventions.” It is reasonable to assume that the human contact provided by research coordinators, provision of ongoing assessments, and reimbursement to incentivize the completion of assessments—none of which are available in real-world use—impact engagement patterns with the interventions. Second, from an external validity perspective, recruitment challenges in trials are often addressed by increasing the reach to potential participants through the expansion of participating venues and the refinement of social media strategies [13]. In this way, researchers unintentionally recruit people who are much more likely to adhere to e-mental health technologies than people in the general population who download and try available programs “in the wild.” Such assumptions are supported by a systematic review of internet interventions for anxiety and depression, which found that the rates of attrition in randomized controlled trials were lower than the reported dropout rates from open-access websites [22].

Overall, there is a need to understand how the general population engages with the most popular unguided mobile apps targeting anxiety, depression, or emotional well-being, and whether there is a difference in how individuals engage with these apps depending on the mental health focus or incorporated techniques. Although some developer-led studies have published results on the use of individual mental health apps deployed in real-world settings, to the best of our knowledge, no study has examined a large sample of mental health apps relying on independently collected data. This investigation is feasible by leveraging the big data commonly generated and stored by digital platforms that record user traffic in the wild [23,24]. Leveraging such data, this examination provides benchmarks of app usage in the real world, where the general public is expected to benefit from their engagement with unguided programs. This information could shed light on specific engagement problems and opportunities for new intervention development and may offer a resource for researchers and developers who want to study and compare their app performance with similar apps.

For this study, a panel provided objective aggregated nonpersonal data on user engagement with mobile apps to analyze patterns of mental health app usage. The three primary aims were to (1) describe common usage patterns of popular unguided apps based on available metrics, (2) identify patterns of user retention over the first 30 days after app installation, and (3) explore whether these patterns differ based on the app’s mental health focus and primary incorporated techniques.

## Methods

### Search Strategy

The search strategy aimed at identifying the most-installed unguided apps targeting depression, anxiety-related problems, or mental health. We used keywords related to depression and anxiety because of the high prevalence of these conditions [25,26]. We also included mental health apps that focused on happiness or the enhancement of mental health (ie, mindfulness meditations) because our previous work identified them as highly popular mental health tools [27,28]. We conducted a systematic engine search of the Google Play Store in November 2018 using the following terms: “depression” OR “mood” OR “anxiety” OR “panic attack” OR “phobia” OR “social phobia” OR “PTSD” OR “posttraumatic stress disorder” OR “stress reduction” OR “worry relief” OR “OCD” OR “obsessive compulsive disorder” OR “mental health” OR “emotional well-being” OR “happiness.” One researcher documented all the apps emerging from the first 100 search results of each keyword, removed duplicates, and sorted them alphabetically. We also included a manual search of apps presented on MindTools.io [27] and PsyberGuide [29].

### App Screening and Inclusion Criteria

#### Determining Apps’ Number of Installs Threshold

To avoid including apps without a representative number of users, and to determine a minimum threshold for inclusion, we assessed the install categories presented by Google Play based on the number of app installs (eg, 10,000, 50,000 installs). Table 1 presents a preliminary analysis of the number of identified apps in each install category and the aggregated minimum number of app installs and corresponding percentages. Included apps had at least 5000 installs after removing any nonrelevant apps based on their title (ie, apps that were clearly not targeted at emotional well-being such as Heart Rate Monitor & Pulse Checker, 7 Minute Workout, 30 Day Fitness Challenge). Adding all the apps in the 5000 installs category would have resulted in a less than 0.5% increase in the total sample of users. Therefore, we determined an inclusion threshold of 10,000 app installs. Table 1 also shows that a small number of apps within the higher install categories were responsible for the most app installs. To make sure that including a large portion of apps with a relatively smaller number of installs (eg, <10,000 app installs) would not bias the results, we also examined whether there was a difference in the pattern of results based on the number of app installs. This will be further explained in the data analysis section.

**Table 1:** Analysis of install categories based on the number of apps in each category.

Install category	Apps identified, n	Minimum identified app installs within this category^a^, n	Cumulative frequency of app installs based on category threshold^b^, n	Added percentage of installs to the overall sample^c^, %
≥10,000,000	2	20,000,000	20,000,000	100.00
5,000,000-9,999,999	6	30,000,000	50,000,000	60.00
1,000,000-4,999,999	21	21,000,000	71,000,000	29.58
500,000-999,999	23	11,500,000	82,500,000	13.94
100,000-499,999	69	6,900,000	89,400,000	7.72
50,000-99,999	33	1,650,000	91,050,000	1.81
10,000-49,999	103	1,030,000	92,080,000	1.12
5000-9999	66	330,000	92,410,000	0.36

^a^The number of apps multiplied by the minimum number of installs based on the install category.

^b^The accumulated number of app installs in all install categories above and including the current install category.

^c^The added percentage of installs to the total sample if the current install category is added to the analysis; it represents the percentage of the total number of app installs within this category divided by the accumulated number of app installs based on the current category threshold.

#### Inclusion and Exclusion Criteria

To be included in this review, apps had to:

1. Be in English;
2. Have at least 10,000 installs documented on Google Play;
3. Focus on mental illness, mental health, or emotional well-being not specifically related to another medical condition (for example, we excluded apps specifically focused on stress reduction due to a physical medical issue such as heart attack); and
4. Incorporate recognized techniques aimed at promoting self-management of mental health problems such as coping with negative symptoms (eg, feeling nervous, loss of energy), achieving positive results (eg, feeling better), or symptom management (eg, mood tracking). We excluded apps focused on the incorporation of sham techniques (see Multimedia Appendix 1 for a definition of sham techniques).

We excluded apps that:

1. Required payment for installation or provided a free trial only for a limited amount of time because it would be expected to bias program usage (free to install apps that included in-app purchases were not excluded);
2. Were therapist-based (eg, telepsychiatry) because the study was focused on unguided interventions; and
3. Were not meant to be used for more than a few times (eg, tests, one-time exposure technique) or were merely magazines.

Two independent reviewers screened the apps based on the inclusion and exclusion criteria. All disagreements were discussed with a third author with reference to the apps until consensus was reached.

### Coding

Two independent reviewers coded the apps’ incorporated techniques based on the following categories: mindfulness/meditation, tracker (including diary or journal), psychoeducation, peer support, and breathing exercise (not exercised as part of a meditation program). These categories were based on previous work done on the therapeutic components of mental health apps [27,30], drawing on the thematic analysis method suggested by Braun and Clarke [31]. The categories were designed to represent nonoverlapping components of potential therapeutic engagement (see Multimedia Appendix 2 for definitions of categories). Although our goal was to identify how specific techniques related to patterns of app use, our metrics did not enable us to differentiate between various techniques incorporated within the same app (ie, we could not tell which parts in the app the users were using). Therefore, we also added a coding of “primary technique” in cases where the app mostly incorporated one technique that was deemed to be the main reason for the app’s use (eg, mindfulness/meditation). It is important to note that this limitation did not enable us to include app features that might influence user engagement but were not identified as a primary incorporated technique. Similarly, it was not feasible to target specific theoretical modalities, such as cognitive behavioral therapy. Because nearly all apps included some components of cognitive behavioral therapy, these were impossible to dismantle given our data.

An app’s mental health focus was determined in the following manner: first, the app’s description had to explicitly state that it targeted people with [mental health focus] and, second, most of the techniques used within the app had to have been built to help users cope with or manage their symptoms directly related to the mental health focus. We grouped apps based on several mental health foci. Under “mental health problems,” we included apps that were focused on supporting people coping with depression, anxiety-related disorders, and emotional difficulties. We also subcoded the app with the terms (a) anxiety-related disorders or (b) depression if the app specifically targeted only one of these aims. (During our coding process, we did not identify another theme for the remaining apps.) Under “happiness,” we included apps that focused on nurturing happiness or general positivity (eg, exercising gratitude, happiness assessment, suggestions for activities nurturing positive feelings), rather than the management of mental health states or problems.

During our coding process, we found a greater ambiguity around the description of apps with a primary incorporated technique of mindfulness/meditation, which leaned more toward enhancing emotional well-being (ie, helping users achieve a positive sense of experience and good mental health), but also aimed at stress reduction. Therefore, we grouped mindfulness and meditation apps separately and did not attribute either of the two mental health foci to them. For this reason, and to enable a proper comparison between categories, we present the mindfulness/meditation category in both the mental health focus and technique outcomes, despite being the same results.

A Cohen kappa interrater agreement of .92 was obtained for coding the variables of interest (incorporated technique, primary technique, and mental health focus). All disagreements were discussed with a third author with reference to the apps until consensus was reached.

### Behavioral Data on User Engagement in the Real World

Information on user traffic was obtained from SimilarWeb’s Pro panel data [32]. The panel provides aggregated nonpersonal information on user engagement with websites and mobile apps all over the world to enable Web and mobile app traffic research and analytics. The panel is based on several sources of anonymized usage data, such as data obtained from consenting users of mobile apps (ie, products). A dedicated product team at SimilarWeb is responsible for building and partnering with hundreds of high-value consumer products that make up the panel. According to SimilarWeb, the products are used across diverse audiences, without cluttering the user with advertisements. While benefiting from the products, users contribute to the panel because they enable the documentation of their online or mobile app usage activities seamlessly and anonymously [32]. The data are not used by SimilarWeb or provided to any third parties for the purposes of marketing, advertising, or targeting of individual subjects. The data-gathering procedures comply with data privacy laws, including the way data are collected, anonymized, stored, secured, and used. These procedures are updated regularly based on evolving data privacy legislation and requirements, such as the European Union’s General Data Protection Regulation [33].

Our examination of data validity was tested and presented in a previous study [28]. An Oath researcher [34] (RW) examined 30 randomly selected mobile apps with data on SimilarWeb and usage data in Oath’s independent records. The researcher examined the correlation between the average number of user sessions per day in the two datasets, finding a very strong Spearman correlation (N=30, *r*=.77, *P*<.001). In our study, we also examined the Spearman correlation between app install categories presented on Google Play (eg, 10,000, 50,000) and the number of downloads documented on SimilarWeb, and found a very strong correlation (N=93, *r*=.81, *P*<.001). These findings suggest a sufficient convergent validity, which is recommended to be above .70 [35].

The study was approved by University of Haifa Institutional Review Board, Haifa, Israel. The measures were set to include data gathered over a 12-month period from August 1, 2017, to July 31, 2018. For each app, available metrics on the panel included app open rate (the average percentage of daily active users out of the total sample of people who currently had the app installed), average number of sessions in a day per daily active user, and average daily minutes of use per daily active user. User 30-day retention included the percentage of users who opened the app each day between day 1 and day 30 out of the number of users who installed and opened the app on day 0. Usage patterns by time were available only for apps with a very large number of users. It was represented by two metrics—average percentage of use per hour (24 hours; eg, 7:00 am, 8:00 am) and per day (7 days; eg, Sunday, Monday)—both calculated based on total app usage.

### Data Analysis

We did not assume a normal distribution of the metrics; therefore, medians and interquartile ranges (IQRs) were used as descriptive statistic measures. In cases in which a category included a small number of apps (n≤5), we used range instead of IQR. To examine differences in usage metrics between apps with different mental health foci or techniques, a Kruskal-Wallis one-way analysis of variance (ANOVA) was performed, followed by Mann-Whitney *U* tests to identify the source of the difference. To examine dependencies in the distribution of categorical values in relevant cases, we used chi-square tests. Most app installs came from a small number of apps with a large number of installs (see Table 1), so we conducted a sensitivity analysis to examine whether including apps with a smaller number of installs would bias the results. Mann-Whitney *U* tests were conducted to compare the distributions of the usage patterns for the top 5 installed apps and the remaining apps from each category presented in the results section (and that included more than five apps). We picked the top 5 apps based on their install category in Google Play. In cases in which several apps “competed” for the fifth place in the same install category, the app with the higher number of downloads (as documented in the SimilarWeb user panel) was chosen.

## Results

### Screening

**Figure 1:**
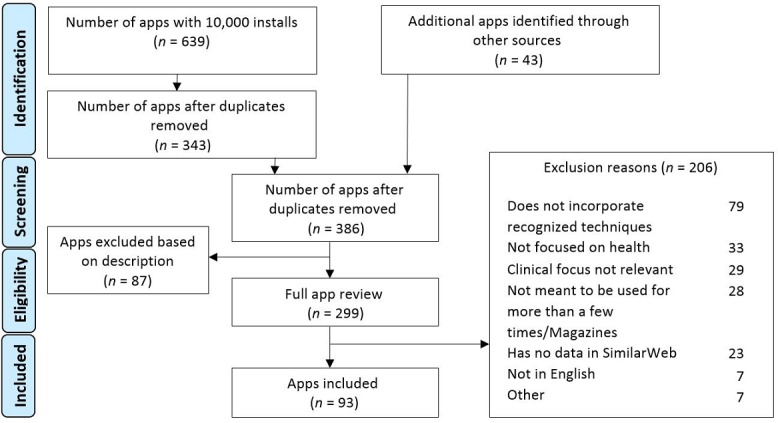
App inclusion flow diagram.

Figure 1 presents the app inclusion flow diagram. The engine search and manual searches produced a total of 386 apps with 10,000 installs or more. Through the first screening process, 299 apps were identified and accessed for a detailed evaluation, and 93 apps were finally included in this study analysis (see Multimedia Appendix 3 for a complete list of included apps).

### Description of Apps

The mental health focus of 59 (63%) apps was a mental health problem. Of these, 19 focused specifically on anxiety-related disorders and 4 focused specifically on depression. In addition, 8 (9%) apps focused on happiness, and 26 (28%) apps focused on the enhancement of emotional well-being through mindfulness/meditation. The distribution of apps based on incorporated techniques is presented in Table 2. Overall, 60 of 93 (65%) apps had a primary incorporated technique, and 33 (36%) apps had two or more incorporated techniques, none of which were primary. Mindfulness/meditation was the most frequent technique as the primary technique of the app (26/93, 28%), followed by use of a tracker (22/93, 24%). Psychoeducation (35/93, 38%) was the most frequent salient technique to be used not as the primary technique, followed by use of a tracker (28/93, 30%).

**Table 2:** Distribution of incorporated techniques in the app sample (N=93).

Incorporated technique	Primary technique, n (%)	Cotechnique^a^, n (%)	Total, n (%)
Mindfulness/meditation	26 (28)	14 (15)	40 (43)
Tracker	22 (24)	28 (30)	50 (54)
Breathing exercise	7 (8)	20 (22)	27 (29)
Psychoeducation	3 (3)	35 (38)	38 (41)
Peer support	2 (2)	7 (8)	9 (1)

^a^The technique is saliently presented in the app but is not considered a primary technique.

### App Usage by Daily Active Users

All apps had complete metrics on app usage by daily active users. Medians and IQRs of daily app usage are presented in Table 3 based on the app’s mental health focus and in Table 4 based on the app’s incorporated techniques. As shown in Table 3, the median app open rate was 4.0% (IQR 4.7%), with medians of 3.28 (IQR 2.53) daily sessions and 13.03 (IQR 14.27) minutes of app use per active user. Daily active usage of mindfulness/meditation apps (median 21.47, IQR 15.00) was found to be significantly higher than the usage of apps for mental health problems (median 10.02, IQR 10.60; *z*=4.64, *P*<.001) or for happiness (median 7.77, IQR 6.90; *z*=3.82, *P*<.001). No other significant difference in app usage was found between mental health foci, including between anxiety- and depression-related apps. As seen in Table 4, the number of app minutes of use was significantly higher for mindfulness/meditation (median 21.47, IQR 15.00) and peer support (median 35.08, n=2) than for other techniques (all *z* ≥2.11, all *P*<.05). In addition, tracker (median 6.3%, IQR 10.2%) and peer support (median 17.0%, n=2) apps had significantly higher open rates than breathing exercise apps (median 1.6%, IQR 1.6%; all *z* ≥3.42, all *P*<.001). No significant differences in usage patterns were found for apps without a primary strategy that incorporated more than one technique.

**Table 3:** App usage based on app mental health focus (N=93).

Mental health focus		Apps, n	Installation category, median (IQR)	Open rate (%), median (IQR)	Daily number of sessions per active users, median (IQR)	Daily minutes of use per active user, median (IQR)^a^
All apps		93	100,000 (90,000)	4.0 (4.7)	3.28 (2.53)	13.03 (14.27)
**Mental health problems**		59	50,000 (90,000)	4.0 (5.1)	3.77 (3.15)	10.02 (10.60)^*^
	Anxiety	19	10,000 (40,000)	2.6 (2.5)	3.58 (3.49)	08.17 (09.42)
	Depression	4	100,000 (50,000-100,000^b^)	4.8 (3.0-6.8^b^)	5.22 (3.97-6.55^b^)	06.97 (02.05-15.12^b^)
Happiness		8	100,000 (50,000)	3.7 (5.3)	3.50 (4.18)	7.77 (6.90)^*^
Mindfulness/meditation^c^		26	100,000 (650,000)	4.1 (3.3)	2.96 (1.66)	21.47 (15.00)^**^

^a^Categories with different number of asterisks (*, **) within a column are significantly different (*P*<.05) based on our analytical approach, which included Kruskal-Wallis one-way ANOVA at the variable level, followed by Mann-Whitney *U* tests.

^b^Due to a small number of included apps, brackets in this cell reflect the range (minimum-maximum value) and not the IQR.

^c^Mindfulness/meditation is presented as a separate mental health focus because all apps in this category were not attributed to another focus as they focus on enhancement of well-being as well as stress reduction.

**Table 4:** App usage based on app incorporated technique (N=93).

Incorporated technique		Apps, n	Installation category, median (IQR)	Open rate (%), median (IQR)^a^	Sessions per active user, median (IQR)	Daily minutes of use per active user, median (IQR)^a^
**Primary technique**						
	Mindfulness/meditation	26	100,000 (650,000)	4.1 (3.3)	2.96 (1.66)	21.47 (15.00)^*^
	Tracker	22	50,000 (90,000)	6.3 (10.2)^*^	4.58 (4.47)	07.27 (08.83)^**^
	Breathing exercise^b^	7	10,000 (40,000)	1.6 (1.6)^**^	2.19 (1.23)	08.32 (19.02)^**^
	Psychoeducation	3	10,000 (10,000-100,000^b^)	3.0 (2.5-3.3^c^)	4.16 (2.57-4.80^c^)	03.53 (02.07-19.23^c^)^**^
	Peer support^d^	2	300,000 (N/A^e^)	17.0 (N/A)^*^	8.67 (N/A)	35.08 (N/A)^*^
**Number of primary techniques**						
	2 techniques	17	50,000 (90,000)	4.0 (5.6%)	3.18 (1.40)	07.83 (11.93)
	≥3 techniques^f^	16	100,000 (50,000)	3.2 (3.1%)	4.06 (3.91)	12.88 (07.13)

^a^Categories with different number of asterisks (*, **) within a column are significantly different (*P*<.05) based on our analytical approach, which included Kruskal-Wallis one-way ANOVA at the variable level, followed by Mann-Whitney *U* tests.

^b^Not including mindfulness/meditation.

^c^Due to the small number of included apps, brackets in this cell reflect the range (minimum-maximum value) and not the IQR.

^d^Due to the small number of included apps, IQR or range could not be calculated (marked with N/A).

^e^N/A: not applicable.

^f^Includes two apps that use a chatbot (Wysa, Woebot), which did not have a different pattern of results emerging for a certain direction.

### User 30-Day Retention

**Figure 2:**
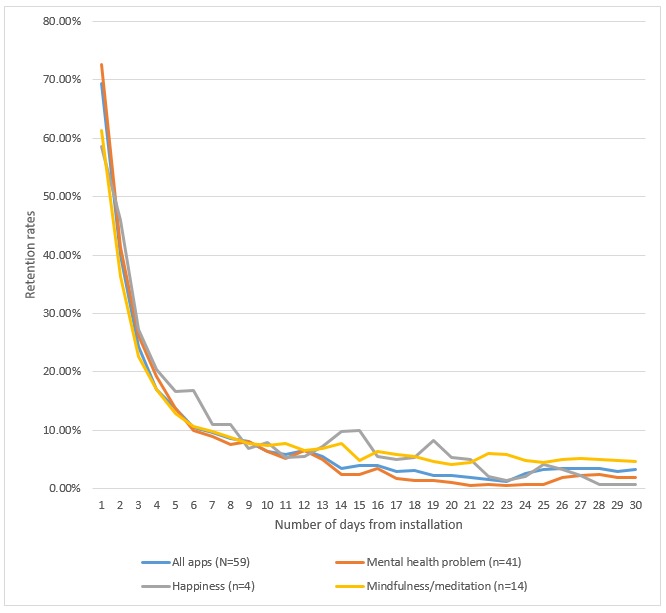
App 30-day retention by mental health focus. The percentages reflect the number of users who opened the app from day 1 to day 30 out of the number of users who installed and opened the app on day 0.

**Figure 3:**
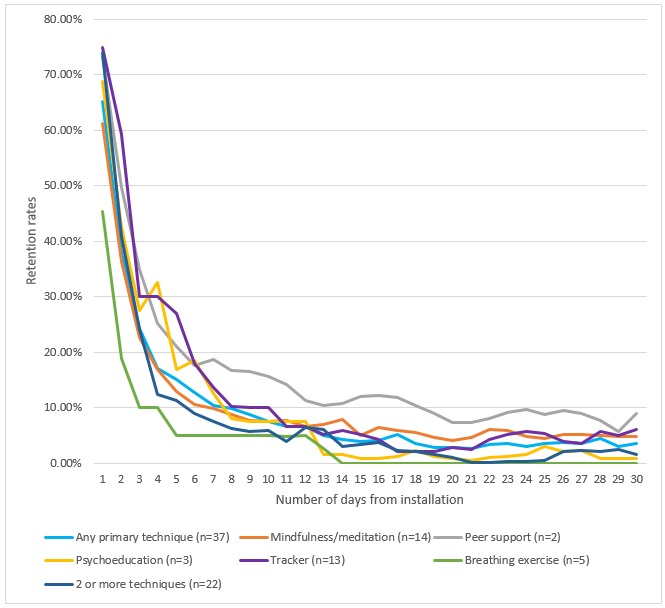
App 30-day retention by primary incorporated technique. The percentages reflect the number of users who opened the app from day 1 to day 30 out of the number of users who installed and opened the app on day 0.

Fifty-nine apps (63%) had data on user retention. Chi-square tests for independence revealed no difference between apps with or without user retention data in the distribution of mental health foci (χ^2^_2_=2.1, *P=*.36) and primary incorporated techniques (χ^2^_4_=3.8, *P*=.44). Figure 2 presents user 30-day retention by the app’s mental health focus; Figure 3 presents user 30-day retention by the app’s incorporated technique. In both figures, there is a sharp decline of more than 80% in app open rates between day 1 and day 10, whereas the differences between day 15 and day 30 are smaller and represent a decline of approximately 20% in app open rates. Figure 2 reveals that, relative to users who opened the app on day 0, the median app open rate was as follows: 69.4% (IQR 27.8%) of users opened it on day 1, 3.9% (IQR 10.3%) of users opened it on day 15, and 3.3% (IQR 6.2%) of users opened it on day 30. Kruskal-Wallis one-way ANOVAs revealed no significant differences in app open rates on day 30 based on mental health focus (H_2_=1.88, *P*=.39) and a significant difference in app open rates on day 30 based on incorporated technique (H_5_=11.31, *P*=.046). Mann-Whitney *U* tests revealed that on day 30 peer support (median 8.9%), mindfulness/meditation (median 4.7%, IQR 6.2%), and tracker/diary apps (median 6.1%, IQR 20.4%) had significantly higher retention rates than breathing exercise apps (median 0.0%, IQR 0.0%; all *z* ≥2.18, all *P* ≤.04). This pattern of difference is also descriptively apparent in 15-day retention, in which the median retention for breathing exercise apps was 0.0% (IQR 0.0%), whereas the range of medians for peer support, mindfulness/meditation, and tracker/diary apps was from 4.9% (IQR 7.1%) to 11.9% (IQR 0.7%).

### Usage Pattern by Hours and Days

**Figure 4:**
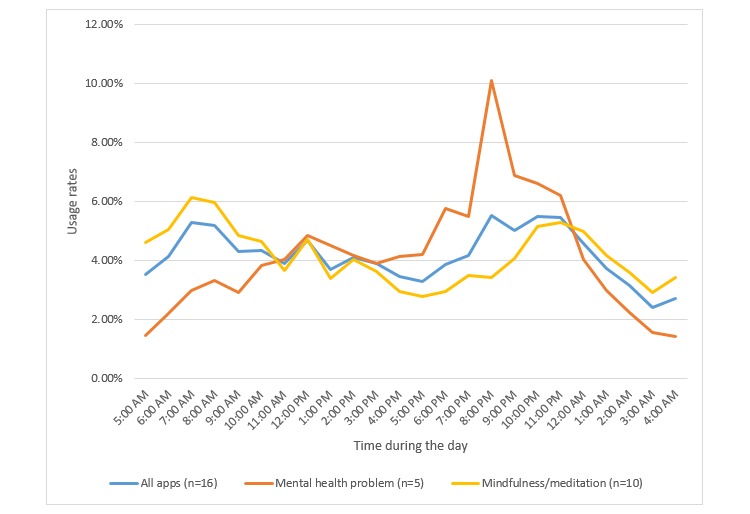
Hourly usage pattern. Usage is presented by hour out of the total app usage; therefore, the sum of percentages within each category is 100%. Note: a subset of apps for which that data were available is included; “All apps” includes both categories and one app targeting happiness.

**Figure 5:**
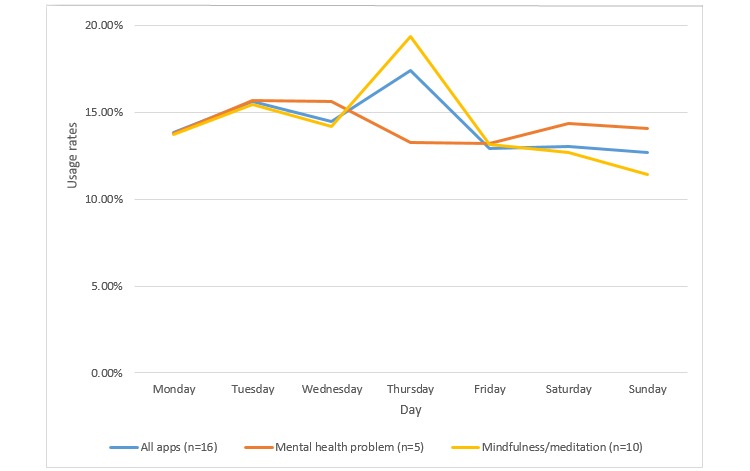
Daily usage pattern. Percentage of app usage is presented by day out of the total app usage; therefore, the sum of percentages within each category is 100%. Note: a subset of apps for which that data were available is included; “All apps” includes both categories and one app targeting happiness.

Sixteen apps had data on hourly and daily app usage. Figure 4 presents the hourly usage patterns of apps and Figure 5 presents the daily usage patterns of apps. The number of apps with available data was small; therefore, we only present categories with data on more than three apps. Furthermore, we have not conducted statistical testing to compare program usage among the different categories. For hourly usage, the results pointed to a peak in app usage in the evening (8:00 pm) for apps targeting mental health problems. The results also showed that mindfulness/meditation apps had two usage peaks: one in the morning (7 am-9 am) and the other in the late evening (10 pm-midnight). In terms of daily usage, the results showed a peak in app usage on Thursday for mindfulness/meditation apps.

### Sensitivity Analysis

We conducted a series of Mann-Whitney *U* tests to examine the difference in app open rate, number of sessions, daily minutes of use, and 30-day retention among the top 5 installed apps and the remaining apps per mental health focus and incorporated technique. We found a significant difference in the open rate of mental health apps favoring the top 5 installed apps (*z*=1.68, *P* ≤.05; top 5 installed apps: median 9.0%, IQR 6.9%; remaining apps: n=54, median 4.0%, IQR 4.7%). Among these five apps, one incorporated online peer support and three incorporated mood trackers. No other differences were found. A series of Mann-Whitney *U* tests was also conducted to examine whether app usage (app open rates, daily number of sessions, daily minutes of use) in each app category (mental health focus, incorporated technique) differed between apps with or without in-app purchases and no significant differences were found (all *P*>.05).

## Discussion

### Principal Findings

This is the first study to report the usage and retention metrics of a large number of frequently installed, unguided mental health apps as recorded “in the wild” and independent of developer-led data. Based on Google Play Store data (using keyword search terms), there were over 90 million mental health app installs documented by the end of 2018 (ie, reach). Although our findings revealed that daily active users use apps for a significant amount of time during the day (daily usage median of 13.03 minutes), most people with the app installed on their device do not open it in any given day (median open rate of 4.0%). Furthermore, general user retention is poor, with a median 15-day retention of 3.9% and 30-day retention of 3.3%. These findings reflect the lower ranges of real-world retention rates reported in developer-led studies [17-20,22].

Our results also indicate that there are significant differences in app usage and user retention that are associated with the app’s incorporated techniques. Daily minutes of use were significantly higher for mindfulness/meditation (median 21.47) and peer support (median 35.08) apps than for apps incorporating other techniques. Daily open rates were significantly lower for breathing exercise apps (median 1.6%) than for apps incorporating the two techniques with the highest open rates (tracker: median 6.3%; peer support: median 17.0%). User 30-day retention was significantly lower for breathing exercise apps (median 0.0%) than for all other incorporated techniques (mindfulness/meditation: 4.7%; trackers: 6.1%; peer support: 8.9%), except for psychoeducation, which exhibited a pattern similar to the breathing exercise apps at 30-day retention. These patterns could be explained using the notion of *effective engagement* described by Yardley and colleagues [36], wherein there is “sufficient engagement with the intervention to achieve intended outcomes.” From this perspective, it might be that once people acquire the desired skills (breathing exercise) or knowledge (psychoeducation) they no longer use the app, thus affecting the pattern of retention over a longer period. By contrast, mindfulness/meditation apps often include guided meditations designed for repeated use over longer periods of time, while not fostering learning or direct skill acquisition.

Our findings on user retention highlight the low engagement with these apps. Although this warrants a re-evaluation of current engagement and retention strategies, it does not necessarily suggest that these apps are only helpful for a small number of users. First, we do not have data implying that users engage only with one app in the self-management of their states or conditions. However, it is difficult to assume that users are knowledgeable about the different apps available, which apps to use, and when to use them. Although there are some recommender websites [27,29,37] and approaches to help users identify the right apps [38-41], a therapeutic framework that provides guidance to users about how to use the right app at the right time could be useful. For example, in their novel study of IntelliCare—a suite of 13 apps and one Hub app accompanied by 8 weeks of coaching to encourage participants to try the apps recommended to them through the Hub app—Mohr and colleagues [42] found that 95% of participants eventually downloaded five or more of the IntelliCare apps as part of their therapeutic process. In another study, patients with schizophrenia spectrum disorders received 6 months of treatment that included health technology coaching around the use of three digital tools that were offered to patients based on their needs; 96% of patients rated the program as beneficial [43]. Future studies are needed to examine the feasibility of executing a scalable framework of care in which users receive the right app recommendation at the right time as part of a self-management routine.

Second, user retention patterns might also indicate the low burden associated with app installation (ie, the simplicity of opening the Google Play Store and clicking the app download and installation buttons), which implies that user context, motivation, and ability to engage [44] with these apps were not tested before app installation. The poor active user rates found in our analysis (median open rates of 4%) suggest that the number of app installs available in app stores do not provide a proper estimation of the proportion of users who actually self-manage their state by using the app. These issues further justify a previous call for the development of models to conceptualize the relationships between user state, need, ability, and motivation to engage with early interventions in the digital public space [8]. Although we need to significantly improve our ability to engage users who have made initial attempts at help-seeking, taking a public health engagement approach that is also focused on sustainability represents an important step forward in scaling effective care.

Finally, we identified that the two apps that incorporated peer support as a primary technique had relatively high engagement and retention rates. In our previous work, we defined a program’s *relatability* as “a good representation of a human factor that is easily relatable within the therapeutic context/process” [38]. Relational factors have also been previously acknowledged to nurture a therapeutic alliance with users [45-47], and have demonstrated to be a quality aspect that predicts user engagement with mobile health interventions [28]. Future studies are needed to determine whether technology has a special advantage as an infrastructure that connects between users and results in better engagement rates.

### Limitations

This study has several limitations that should be considered. First, because we used an anonymous user panel, we did not have data about how different users use the apps and which parts of the apps were more engaging. The absence of such data means that some apps might have been more engaging due to the characteristics of their users, a phenomenon suggested previously by Ernsting and colleagues [48]. In addition, due to this limitation we were only able to focus on primary incorporated techniques within the apps and not on the way different design features (not deemed to be a primary technique) may have impacted the results. Subsequently, because we were leaning on off-the-shelf programs available to the public, we could not manipulate the programs themselves to account for aspects which lacked variability in our data, such as the impact of theoretical modalities on usage. That is, although our study advantage is that it enables us to present benchmarks of real-world use independent to trial settings, one advantage of direct experiments is the ability to control participant identity and manipulate intervention modalities and features to identify the group of active components leading to the best outcome (eg, [49]). Such experiments could be also helpful in determining causal relationships between intervention modalities and user behaviors, based on the context of use.

Second, some techniques such as peer support were only incorporated by a small number of highly installed apps (median installation category of 300,000). However, our results did not indicate a significant difference in any incorporated technique in terms of app installs, which suggests that these apps usage patterns go beyond an app’s popularity.

Third, because we were led by the available metrics on the platform, we could not examine retention rates after the first 30 days. The retention slope presented a slower decline in app open rates between day 15 and 30 and, based on previous reports, it would be reasonable to assume that there is a continuous usage decline over time (eg, [19,50]), but more studies are needed to determine the magnitude of the decline.

Finally, this study was only based on Android users. Current estimates suggest that the Android market share is approximately 88% of mobile phone users globally [51] and approximately 42.7% of mobile phone users in the United States [52]. Although these data suggest that a sufficient portion of users use the Android operating system, it would be beneficial to validate these results with datasets from the Apple market.

### Conclusions

The use of digital platforms that record user traffic “in the wild” enables us to examine patterns of app usage outside of study settings and to assess real-world public engagement. Although we found daily active minutes of use to be relatively high, only a small portion of users actually used popular apps regularly. More studies leveraging different datasets are needed to understand these phenomena. On a broader level, findings point to the importance of the ways we measure, report, and address aspects of user engagement in the real world. It would be helpful to track the context of users who eventually use apps, hopefully through the use of digital footprints, while also tracking the use of multiple apps and websites across times. Obviously, aspects that relate to security and privacy of data have to be addressed. In addition, new studies are needed to better conceptualize our understanding of users’ contexts and the ways they search for and engage with beneficial services outside of traditional health care settings.

## Appendix

Multimedia Appendix 1 Definition of sham techniques.

Multimedia Appendix 2 Definition of coded techniques.

Multimedia Appendix 3 List of included apps.

## Abbreviations

ANOVA: analysis of variance
IQR: interquartile range
