# Supplementary material for: Objective User Engagement With Mental Health Apps: Systematic Search and Panel-Based Usage Analysis
Source: J Med Internet Res. 2019 Sep 25;21(9):e14567. doi: 10.2196/14567 (PMC6785720; doi:10.2196/14567)
Supplement: Multimedia Appendix 1 [file jmir_v21i9e14567_app1.pdf]

Appendix 1 – Definition of Sham Techniques

In our review process we were focused on the exclusion of apps incorporating sham techniques in order to avoid the possibility that poor program usage was the result of the provision of non-appropriate material. Sham techniques were techniques that had no- or very little-research support (e.g., no research relating on studies with low quality of methodological standards). For example, apps focused on the incorporation of coloring pages (i.e. digital coloring) or casual games (e.g. puzzles) aimed for people with anxiety.

Similar to previous reviews of mental health apps (Aguilera & Muench, 2012; Sucala et al., 2017) we defined recognized techniques as evidence based psychological techniques aimed at supporting the user's therapeutic process. Recognized techniques had to have supportive research evidence in relation to mental health interventions, such as the provision of *appropriate* and *accurate* psychoeducation materials (e.g. Donker et al. 2009). This includes the provision of relevant content such as the incorporation of cognitive behavioral therapy principles in an app focusing on anxiety related disorders.

Two independent reviewers screened the apps. All disagreements were discussed with a third author with reference to the apps until consensus was reached.

References

Aguilera, A., & Muench, F. (2012). There's an app for that: Information technology applications for cognitive behavioral practitioners. The Behavior therapist/AABT, 35(4), 65.

Donker, T., Griffiths, K. M., Cuijpers, P., & Christensen, H. (2009). Psychoeducation for depression, anxiety and psychological distress: a meta-analysis. BMC medicine, 7(1), 79.

Sucala, M., Cuijpers, P., Muench, F., Cardoș, R., Soflau, R., Dobrean, A., ... & David, D. (2017). Anxiety: There is an app for that. A systematic review of anxiety apps. Depression and anxiety, 34(6), 518-525.
