# Supplementary material for: Objective User Engagement With Mental Health Apps: Systematic Search and Panel-Based Usage Analysis
Source: J Med Internet Res. 2019 Sep 25;21(9):e14567. doi: 10.2196/14567 (PMC6785720; doi:10.2196/14567)
Supplement: Multimedia Appendix 2 [file jmir_v21i9e14567_app2.pdf]

Appendix 2 – Definition of Coded Techniques

Psychoeducation

Providing education and information to users about mental health and emotional well-being in order to help them better understand and cope with their condition. The process includes, but is not limited to, describing the challenges and causes of the relevant condition, introducing coping strategies, and providing information on internal and external resources.

Tracker

Enables users who want to keep track (based on set or flexible time intervals) of key self-reported aspects that relate to their mental health or emotional well-being – mainly in order to identify patterns over time. These variables could include mood states, symptoms, feelings, behaviors, daily events, context (when and where the user felt this way), and journal (a space to write down diary notes).

Peer Support

Help and support provided through lay people or people who have had similar experiences as the user (e.g., lived experience of a mental illness). The support could be provided, for example, through chat or messaging services (synchronous and asynchronous) or through forums in which users discuss mutual areas of interest around mental health and emotional well-being.

Mindfulness/Meditation

A technique focused on helping people to practice meditation. Forms of meditations help to train users in awareness and to develop a healthy sense of perspective and concentration. The technique includes audio and sometimes video visualizations in which the narrator guides the user through the meditation cycle. This can sometimes be tailored to user expertise in practicing meditation.

Breathing Exercise

Teaching users to control their breathing primarily in order to lower stress by sending a message to the brain that calms and relaxes the body and affects the user's physical state (e.g., lowers heart rate). These exercises are not presented as part of a meditation routine.
