# Supplementary material for: Objective User Engagement With Mental Health Apps: Systematic Search and Panel-Based Usage Analysis
Source: J Med Internet Res. 2019 Sep 25;21(9):e14567. doi: 10.2196/14567 (PMC6785720; doi:10.2196/14567)
Supplement: Multimedia Appendix 3 [file jmir_v21i9e14567_app3.pdf]

Appendix 3 - List of Included Apps

# of Installations	App name	Company/Developer
10,000,000	Headspace: Meditation & Mindfulness	Headspace, Inc.
5,000,000	Calm - Meditate, Sleep, Relax	Calm.com, Inc.
5,000,000	Daylio - Diary, Journal, Mood Tracker	Daylio
1,000,000	Insight Timer - Free Meditation App	Insight Network Inc
1,000,000	Meditate ॐ OM	PANAGOLA
1,000,000	Prana Breath: Calm & Meditate	Oleksandr Albul
1,000,000	Stop, Breathe & Think: Meditation & Mindfulness	Stop, Breathe & Think
500,000	7 Cups: Anxiety & Stress Chat	7 Cups of Tea
500,000	Abide: Christian Guided Meditation & Daily Prayers	Carpenters Code Inc.
500,000	Let's Meditate: Guided Meditation	Heal Me Team
500,000	Meditation & Relaxation: Guided Meditation	Fitness22
500,000	Meditation Game	Being
500,000	Pacifica - Stress & Anxiety	Pacifica Labs Inc.
500,000	Relax Lite: Stress Relief	Saagara
500,000	Self-help Anxiety Management	University of the West of England
500,000	Simple Habit Meditation	Simple Habit, Inc
500,000	The Mindfulness App: relax, calm, focus and sleep	MindApps
100,000	10% Happier: Meditation for Fidgety Skeptics	10% Happier
100,000	21-Day Meditation Experience	Chopra Enterprises LLC
100,000	365 Gratitude: Journal, Grateful Community	UofHappy, LLC
100,000	Aura: Mindfulness & Happiness	Aura Health
100,000	Aware: Meditation & Mindfulness	Zoojoo.be
100,000	Booster Buddy	Island Health
100,000	Breathe2Relax	T2
100,000	Calm Harm - manages self harm	stem4
100,000	CBT Diary	Continuum
100,000	Cognitive Diary CBT Self-Help	Excel At Life
100,000	Depression CBT Self-Help Guide	Excel At Life
100,000	Free Meditation - Take a Break	Meditation Oasis
100,000	Gratitude Journal	John De Mott
100,000	Habit - Success, Prosperity & Happiness.	App Visionaire
100,000	Happify	Happify, Inc.
100,000	Happy Habits: Choose Happiness	Excel At Life
100,000	InnerHour - Self Help for Anxiety & Depression	InnerHour
100,000	Mindfulness: Guided Meditation for Stress, Anxiety	Pedby Wellness Studio
100,000	MindShift™	Anxiety Disorders Association of British Columbia
100,000	Moodpath - Depression & Anxiety	Moodpath

# of Installations	App name	Company/Developer
100,000	MoodSpace	Boundless
100,000	MoodTools - Depression Aid	MoodTools
100,000	Moodtrack Social Diary	Matthew Windwer
100,000	Omvana: Meditation, Yoga Sleep	Mindvalley LLC
100,000	Qi Gong Meditation Relaxation	Excel At Life
100,000	Smiling Mind	Smiling Mind
100,000	Stop Panic & Anxiety Self-Help	Excel At Life
100,000	SuperBetter	SuperBetter, LLC
100,000	T2 Mood Tracker	T2
100,000	TalkLife	TalkLife
100,000	What's Up? - Mental Health App	Jackson Tempra
100,000	Wysa - AI therapy for stress, anxiety & depression	Touchkin
100,000	Year in Pixels	Teo Vogel
100,000	Youper - Anxiety & Depression	Youper, Inc
50,000	aiMei - Personality Tests & Mood Tracking	BPU Holdings
50,000	Anxiety Test	MoodTools
50,000	Breathing exercises	Zarko Petrovic
50,000	Buddhify - mindfulness meditation on the go	Mindfulness Everywhere
50,000	CBT Thought Record Diary	MoodTools
50,000	Dare - Break Free From Anxiety	BMD Publishing
50,000	Deep Meditation: Relaxation & Sleep Meditation App	Deep Relax & Meditation
50,000	Depression Test	MoodTools
50,000	Gratitude journal - private diary & daily quotes	Pritesh Sankhe
50,000	Jealousy Test & CBT Self-Help	Excel At Life
50,000	Kardia - Breath Stress Relief	Labna Sounds
50,000	Mental Health Tests	Mind Diagnostics, Inc.
50,000	Mindfulness Coach	US Department of Veterans Affairs (VA)
50,000	PTSD Coach	US Department of Veterans Affairs (VA)
50,000	Wildflowers Mindfulness	Mobio Interactive Inc.
10,000	ACT Companion: The Happiness Trap App	Berrick Psychology
10,000	Anti Anxiety - Depression, Stress, Panic Attack	App Visionaire
10,000	Breath Ball: The Stress Relief Breathing Exercise.	Fun Driven
10,000	Breathe Easy	MoodTools
10,000	Catch It	The University of Liverpool
10,000	Cognitive Behavioral Therapy: Depression & Anxiety	NKDigital
10,000	FearTools - Anxiety Aid	MoodTools
10,000	Flowy Beta	Playlab London
10,000	How Are You? - Mood tracker	Psykolog Lene Bammeskov

# of Installations	App name	Company/Developer
10,000	iMoodJournal	Inexika Inc.
10,000	MindCare: mental well-being analytics made easy	Blue Bird Technologies
10,000	Mood Log	AR Productions Inc.
10,000	Mood Triggers: Anxiety Depression Insomnia Tracker	Mood Triggers: Anxiety & Depression Tracker
10,000	MoodMission - Cope with Stress, Moods & Anxiety	MoodMission Pty Ltd
10,000	Panic Attacks?	ilovepanicattacks
10,000	PanicShield - Panic Attack Aid	MoodTools
10,000	Rootd - Panic Attack & Anxiety Relief	Simply Rooted Media
10,000	Social Anxiety Test	MoodTools
10,000	Stories – Timeline Diary / Journal, Mood Tracker	Hadi Satrio
10,000	Stress Management	ap developers
10,000	Tactical Breather	T2
10,000	The Anxiety Guy Podcasts - Fear, Depression Relief	Pitashi Mobile Imagination
10,000	The Happiness Planner	BRANDMENTALIST LLC
10,000	Thought Challenger	CBITs
10,000	TruReach - Anxiety, Stress & Depression	TruReach Health
10,000	Woebot - CBT for Stress, Anxiety & Depression	Woebot Labs
10,000	Worry Knot	CBITs
